# Myocardial Injury in Hospitalized Patients with Myasthenia Gravis

**DOI:** 10.3390/jcm11237106

**Published:** 2022-11-30

**Authors:** Hongxi Chen, Lingyao Kong, Ying Zhang, Xue Lin, Ziyan Shi, Qin Du, Xiaofei Wang, Yanlin Lang, Linjun Cai, Zichao Mou, Wenqin Luo, Shuangjie Li, Hongyu Zhou

**Affiliations:** Department of Neurology, West China Hospital, Sichuan University, Chengdu 610041, China

**Keywords:** myocardial injury, myasthenia gravis, thymoma, infection, myasthenic crisis, immune checkpoint inhibitors

## Abstract

Objective: To investigate the clinical characteristics and outcome of myocardial injury in patients with myasthenia gravis (MG). Methods: We retrospectively searched medical records to screen hospitalized patients with MG at our hospital. The troponin T (TnT) levels were deemed necessary to be performed based on the patient’s clinical symptoms and were used as biomarkers of myocardial injury. The patients’ demographic and clinical information were collected. Death was the primary outcome. Results: A total of 336 patients with MG measured TnT levels and were included in the final analysis. The male MG patients with elevated TnT levels had a higher prevalence of infection (56.8% vs. 30.0%, *p* = 0.001) and myasthenic crisis (37.5% vs. 13.3%, *p* = 0.001) than those with normal TnT levels. Meanwhile, the female MG patients with elevated TnT levels were older (56.0 (16.6) vs. 49.2 (17.2)) years old, *p* = 0.007] and had a higher prevalence of infection (65.4% vs. 32.1%, *p* < 0.001), myasthenic crisis (33.6% vs. 17.9%, *p* = 0.015), and thymoma (38.5% vs. 16.7%, *p* = 0.001) than those with normal TnT levels. Older age (coef. = 0.004; *p* = 0.034), infection (coef. = 0.240; *p* = 0.001), myasthenic crisis (coef. = 0.312; *p* < 0.001), thymoma (coef. = 0.228; *p* = 0.001), and ICI therapy (coef. = 1.220; *p* < 0.001) were independent risk predictors for increasing log TnT levels. Thirty-seven patients died during hospitalization. High log TnT levels (OR = 8.818; *p* < 0.001), female sex (OR = 0.346; *p* = 0.023), thymoma (OR = 5.092; *p* = 0.002), and infection (OR = 14.597; *p* < 0.001) were independent risk predictors of death. Conclusions: Our study revealed that the surveillance of myocardial injury biomarkers in MG patients might be beneficial.

## 1. Introduction

Myasthenia gravis (MG) is an autoimmune neuromuscular disease characterized by skeletal muscle weakness, and it is most commonly mediated by autoantibodies that target the acetylcholine receptor (AChR). The patient’s weakness can be generalized or localized and is typically exacerbated with exercise and repetitive muscle use [1]. It is well known that skeletal muscle involvement is always responsible for the weakness from MG.

However, the cardiac dysfunction was also associated with MG. Dysfunction of the cardiac autonomic nervous system with predominant parasympathetic impairment might lead to abnormal baroreflex sensitivity, as well as heart-rate and blood-pressure variability [2,3,4]. Recently, myocardial injury was also reported in patients with MG [5]. The Fourth Universal Definition of Myocardial Infarction defines myocardial injury as cardiac troponin concentrations that are above the 99th percentile upper reference limit (URL). Patients with dynamic changes have acute injury, and those patients without changes have chronic injury [6]. Myocarditis is a common nonischaemic cause of myocardial injury and is considered an inflammatory disease of the heart that may occur as a consequence of infection, exposure to toxic substances, and immune system activation [7,8].

Although a small fraction of cardiac troponin may emanate from injured skeletal muscle [9], cardiac troponin is generally regarded as a biomarker for the diagnosis of myocardial injury and is recommended for routine clinical use or heart-related studies [10,11]. Recently, a systematic review suggested that nearly half of the patients with MG complicated with myocarditis died during hospitalization. That review only included 35 patients from 28 studies and had a high risk of selection bias. Despite this, we were reminded to provide deep insight into the involvement of myocardial injury in MG patients. Although the exact mechanism of myocardial injury in MG was still unclear, the well-known striational antibodies, which bind to both skeletal muscle cells and cardiomyocytes, are one of the most plausible explanations [5].

In the present study, we used cardiac troponin T (TnT) as a biomarker of myocardial injury and aimed to investigate the clinical characteristics and outcome of nonischaemic myocardial injury in patients with MG.

## 2. Methods

### 2.1. Study Design and Patient Selection

This study was approved by the Medical Ethics Committee of the West China Hospital of Sichuan University. We retrospectively searched all inpatient medical records for patients who had a diagnosis of MG at the West China Hospital of Sichuan University from January 2011 to September 2021. The typical clinical manifestation was the primary basis for the diagnosis of MG, including partial or generalized striated muscle fatigue, which was aggravated after exercise and improved after rest. Meanwhile, the final diagnosis of MG required a positive serologic test for autoantibodies; a positive neostigmine test; or abnormal repetitive nerve stimulation [1]. We excluded patients with coexisting heart disease, such as ischaemic myocardial injury, and patients with an estimated glomerular filtration rate (eGFR) < 60 mL/min/1.73 m^2^.

### 2.2. Data Acquisition

The data collected from the medical records included demographic data (age and sex), cardiac TnT levels, creatinine levels, infection and myasthenic crisis during hospitalization, and outcome (death). We also collected other clinical information, such as classification (ocular or generalized), treatment, chest computerized tomography (CT), and thymus pathology.

**Figure 1:**
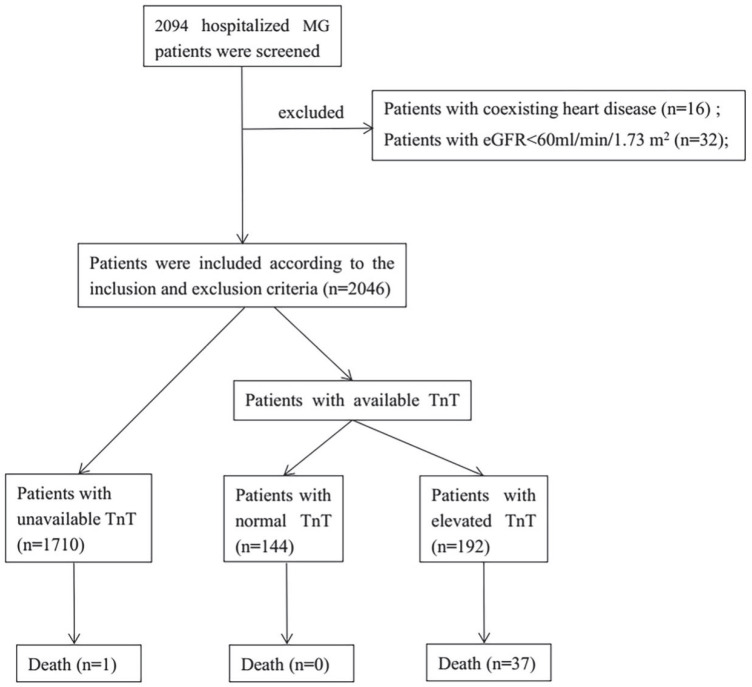
Flowchart of the selection of patients with MG. Abbreviations: MG, myasthenia gravis; eGFR, estimated glomerular filtration rate; and TnT, Troponin T.

Blood samples were analyzed with a biochemistry analyzer at the Medical Laboratory of West China Hospital. eGFR was evaluated according to the equation of the 2009 Chronic Kidney Disease Epidemiology Collaboration [12]. The reference range for cardiac TnT levels was 0–14 ng/L. Myocardial injury was defined as blood levels of cardiac TnT above 14 ng/L, regardless of new abnormalities on electrocardiography and echocardiography. The TnT levels were deemed necessary to be performed based on the cardiac-like clinical symptoms, such as chest pain, chest tightness, dyspnoea, fatigue, palpitations, or syncope. The changes on the electrocardiogram, echocardiography, and coronary angiography were used to exclude coexisting heart disease, such as ischaemic myocardial injury. The patients were grouped based on the following: elevated TnT, normal TnT, and unavailable TnT levels (Figure 1).

The presence of infection was defined according to the diagnosis in the medical records, and the infections included respiratory tract infections, urinary tract infections, gastrointestinal infections, intracranial infections, and septic shock. Myasthenic crisis was defined as worsening of respiratory muscle weakness requiring intubation or noninvasive ventilation to avoid intubation. Chest CT was performed using 1 to 5 mm thick slices with the commercial multidetector CT scanners in our hospital. The diagnosis of thymoma was made on the basis of chest CT and confirmed by pathologic examination.

### 2.3. Statistical Analyses

Quantitative data are described as the mean (standard deviation (SD)). Categorical data were described as percentages. Differences in normally distributed data were tested using Student’s *t* test, and Pearson’s chi-squared test (χ^2^) was performed to the categorize variables.

Because the test of normality showed that the TnT levels were not normally distributed, a log transformation was applied to the variant to improve the normality of the data. To determine the associations between the demographic and clinical characteristics and the log TnT levels, univariate and adjusted multivariate linear regression models were performed for the patients with available TnT levels. Moreover, the logistic regression models were performed to explore the correlation of death and the log TnT levels. Coefficients (coef.) or odds ratio (OR) with their 95% confidence intervals (CIs) were calculated. All statistical analyses were performed using STATA 17.0 (StataCorp., College Station, TX, USA). A 2-sided *p* value < 0.05 was considered statistically significant.

## 3. Results

### 3.1. Baseline Characteristics

A total of 2094 hospitalized patients with MG were screened. We excluded 16 patients with coexisting heart disease and 32 patients with an eGFR < 60 mL/min/1.73 m^2^. Finally, 1710 patients with MG did not measure TnT levels and were excluded in the following analysis. TnT levels were available in 336 patients with MG, and 192 of the patients showed elevated TnT levels (Figure 1). None of them were diagnosed as the Coronavirus Disease 2019, based on the detection of chest CT, antibodies, or the nucleic acid of the novel coronavirus.

**Figure 2:**
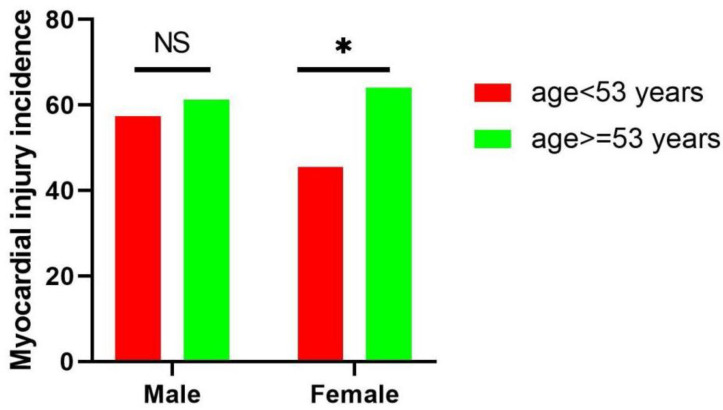
Age and sex-group-specific incidence rate of elevated TnT levels (myocardial injury). * *p* < 0.05. NS, non-significant.

The baseline characteristics of the included patients are displayed in Table 1. The male MG patients with elevated TnT levels had a higher prevalence of infection (56.8% vs. 30.0%, *p* = 0.001) and myasthenic crisis (37.5% vs. 13.3%, *p* = 0.001) than those with normal TnT levels. Meanwhile, the female MG patients with elevated TnT levels were older (56.0 (16.6) vs. 49.2 (17.2) years old, *p* = 0.007) and had a higher prevalence of infection (65.4% vs. 32.1%, *p* < 0.001), myasthenic crisis (33.6% vs. 17.9%, *p* = 0.015), and thymoma (38.5% vs. 16.7%, *p* = 0.001) than those with normal TnT levels. However, the prevalence of generalized MG (male: 82.6% vs. 71.2%, *p* = 0.105; female: 91.0% vs. 81.9%, *p* = 0.070), atrial fibrillation (male: 1.1% vs. 0%, *p* = 0.407; female: 3.9% vs. 0%, *p* = 0.069), and taking immune checkpoint inhibitors (ICIs) (male: 3.4% vs. 1.7%, *p* = 0.521; female: 2.9% vs. 0%, *p* = 0.117) was not significantly different between patients with elevated and normal TnT levels. (Table 1, Figure 2) According to the echocardiographic measurements, the left ventricular end-diastolic diameter was smaller in female patients with elevated TnT levels than in those with normal TnT levels (42.9 (3.4) vs. 44.6 (3.2) millimeters, *p* = 0.017). The other cardiac structure was similar between patients with elevated and normal TnT levels (*p* > 0.05) (Table 2).

### 3.2. Myocardial Injury Examinations of Patients with MG

**Figure 3:**
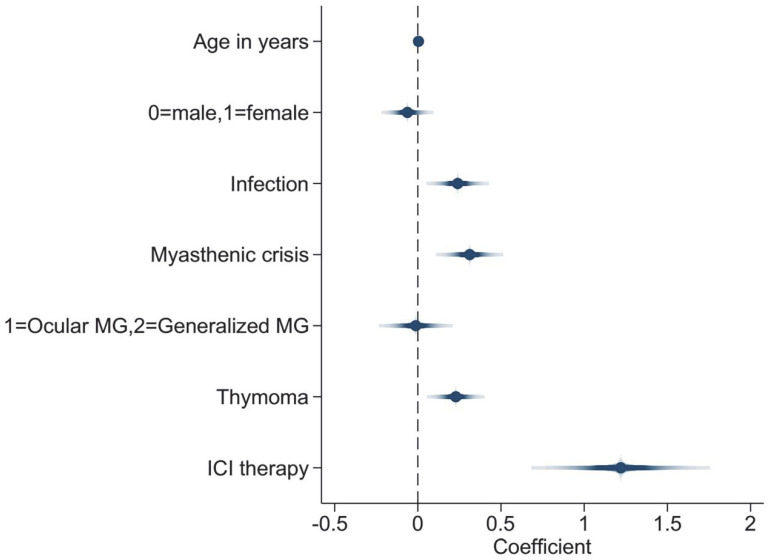
Coefplot showing the adjusted multivariate linear regression models with log Troponin T levels as the dependent variable. Abbreviations: MG, myasthenia gravis; ICI, immune checkpoint inhibitor.

In Table 3, the multivariate linear regression model for the patients with available TnT levels (n = 336) showed that older age (coef. = 0.004; 95% CI: 0.000, 0.007; *p* = 0.034), infection (coef. = 0.240; 95% CI: 0.097, 0.382; *p* = 0.001), myasthenic crisis (coef. = 0.312; 95% CI: 0.158, 0.466; *p* < 0.001), thymoma (coef. = 0.228; 95% CI: 0.097, 0.360; *p* = 0.001), and ICI therapy (coef. = 1.220; 95% CI: 0.812, 1.628; *p* < 0.001) were independent risk predictors for increasing log TnT levels. Nevertheless, sex (coef. = −0.063; 95% CI: −0.182, 0.056; *p* = 0.301) and classification (ocular or generalized; coef. = −0.012; 95% CI: −0.181, 0.158; *p* = 0.888) were not significantly associated with log TnT levels. (Table 3, Figure 3).

### 3.3. Outcomes

Thirty-seven patients with available TnT levels died during hospitalization, and TnT levels were elevated in all of them (Figure 1). The characteristics of the 37 dead patients (average age, 58.2 (16.0) years; female, 43.2%) are displayed in Table 4. The prevalence of infection and thymoma was 91.9% and 51.4%, respectively. Two of the patients were taking ICIs. Moreover, all of the patients who died were generalized MG and complicated with myasthenic crisis (Table 4).

**Figure 4:**
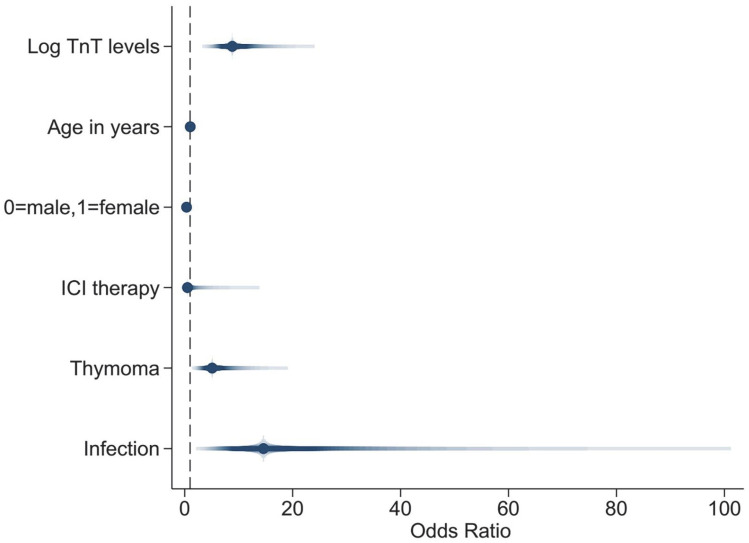
Coefplot showing the adjusted multivariate logistic regression models with death as the dependent variable.Abbreviations: TnT, Troponin T; ICI, immune checkpoint inhibitor.

Furthermore, we included the potential demographic and clinical predictors in the adjusted logistic multivariate analysis, and the results showed that high log TnT levels (OR = 8.818; 95% CI: 4.107, 18.934; *p* < 0.001), female sex (OR = 0.346; 95% CI: 0.139, 0.862; *p* = 0.023), thymoma (OR = 5.092; 95% CI: 1.861, 13.938; *p* = 0.002), and infection (OR = 14.597; 95% CI: 3.345, 63.700; *p* < 0.001) were independent risk predictors of death. Age (OR = 1.028; 95% CI: 0.994, 1.063; *p* = 0.112) and ICI therapy (OR = 0.514; 95% CI: 0.042, 6.308; *p* = 0.603) were not significantly associated with death. (Table 5, Figure 4)

## 4. Discussion

Examination of the serum TnT is inexpensive and widely available, and elevated TnT levels generally indicate myocardial injury. Previous studies suggested that myocardial injury should be considered a component of MG^5^. However, the findings from several case reports indicate low-grade evidence. In the present study, the decision to perform examinations of the TnT levels was based on the patient’s clinical symptoms. Finally, the TnT levels were available in 336 patients. These results suggested that myocardial injury is not rare in MG. Additionally, we found that the MG patients with elevated TnT levels were older and were more frequently complicated with infection, myasthenic crisis, and thymoma than those with normal TnT levels. Older age, infection, myasthenic crisis, thymoma, and ICI therapy were independent risk predictors of myocardial injury. After adjusting for age, sex, ICI therapy, thymoma, and infection, myocardial injury was an independent risk predictor of death during hospitalization. This reminds us that careful attention should be paid to myocardial injury in patients with MG, especially those with older age, infection, myasthenic crisis, thymoma, and ICI therapy.

Consistent with previous studies [13,14], our study showed that the presence of thymoma was an independent risk predictor of myocardial injury and death in patients with MG. The mechanism underlying the association between thymoma and myocardial injury in MG patients remains uncertain. One of the most plausible explanations is as follows: thymoma always contributes to the formation of an excessive amount of autoantibodies. Antibodies that bind to both skeletal muscle cells and cardiomyocytes, known as striational antibodies, have been detected [15]. The representative striational antibodies against striational proteins include titin, ryanodine receptors, and muscular voltage-gated potassium channel (VGKC) Kv1.4. Among them, anti-Kv1.4 is the most widely studied. S. Suzuki et al. reported that 70 of 650 (10.8%) MG patients had anti-Kv1.4 antibodies. It is well recognized that Kv1.4 is essential to maintain cardiomyocyte function [16]. Suzuki et al. reported that myocarditis was clinically suspected in eight patients with anti-Kv1.4 antibodies but was not found in any of the patients without anti-Kv1.4 antibodies [17]. This suggests that the anti-Kv1.4 antibody may be a possible biomarker for heart involvement in patients with MG. Moreover, a greater proportion of anti-Kv1.4 was detected in patients with thymoma-associated MG (40–70%) [15]. Therefore, thymoma may increase the formation of striational antibodies, such as anti-Kv1.4, thereby causing myocardial injury.

Most importantly, in the 2046 included patients with MG, 38 died. In addition, 37 of them had elevated TnT levels. The logistic regression model also showed that myocardial injury independently increased the risk of in-hospital mortality. A recent systematic review suggested that nearly half of the patients with MG complicated with myocarditis died during hospitalization [5]. These results indicated a close association between myocardial injury and death. However, patients with myocardial injury may be asymptomatic or could present with nonspecific symptoms, such as chest tightness, dyspnoea, and fatigue [5], which are sometimes difficult to differentiate from MG-like symptoms. Thus, clinicians are always prone to overlook myocardial injury, thereby leading to delayed diagnosis and poor prognosis. Therefore, early recognition and early treatment of myocardial injury are critical.

In our cohort, thymoma and infection were independent risk predictors of death. There is a strong association between thymoma and MG [1]. Thymoma-associated MG patients had more severe myasthenic symptoms, a worse prognosis, and higher mortality [18]. Moreover, studies have revealed that patients with MG and thymoma might be prone to developing myocardial injury [13,14]. Infection is a frequent phenomenon and a common cause of death in patients with MG [19,20]. Infectious disease is also the most common noncardiac reason for myocardial injury [6]. Infectious disease may induce inflammatory cells to infiltrate into the myocardium and cause inflammatory cardiomyopathy [6,21]. This was consistent with our present study, which showed that infection was more common in MG patients with myocardial injury. Furthermore, infection was a strong trigger of a myasthenic crisis. Takotsubo cardiomyopathy triggered by myasthenic crisis might also contribute to myocardial injury and death [22]. Therefore, surveillance of myocardial injury biomarkers is of great importance in MG patients complicated with thymoma, infection, and myasthenic crisis. Additionally, the mortality rate of the patients with normal TnT levels was obviously lower than that of the patients with elevated TnT levels. This result suggested that although cardiac-like clinical symptoms do not necessarily indicate myocardial injury, close monitoring of TnT is important.

Many patients with chronic kidney disease have elevated TnT levels due to small-vessel coronary obstruction, hypotension, and possibly direct toxic effects on the myocardium associated with a uraemic state [6]. To minimize the effect of kidney disease, patients with abnormal kidney function (eGFR < 60 mL/min/1.73 m^2^) were excluded from the present study. Further research is needed to study populations with abnormal kidney function.

In conclusion, myocardial injury associated with MG is not unusual; however, clinicians are always prone to overlook myocardial injury. Many patients were not screened for myocardial injury, especially patients whose symptoms were nonspecific or asymptomatic. On the other hand, myocardial injury was an independent risk predictor of death. Nearly half of the patients with MG complicated with myocarditis died during hospitalization [5]. Therefore, it is essential to pay more attention to myocardial injury in patients with MG.

Several limitations of this study should be addressed. First, we only included hospitalized patients. The patients in the inpatient department had more severe disease than those in the outpatient department. Not all of the hospitalized MG patients underwent myocardial injury examinations. Thus, unknown factors that affect the serum TnT levels might be overlooked. Second, we could not differentiate all the reasons related to myocardial injury. Endomyocardial biopsy and cardiac magnetic resonance imaging were not available. Although we excluded patients with ischaemic myocardial injury, confounding factors such as viral myocarditis and Takotsubo cardiomyopathy were not eliminated. Third, the serum TnT levels were the only biomarker of myocardial injury in the present study. A small fraction of cardiac troponin may be released from injured skeletal muscle. This may lead to a bias in the diagnosis of myocardial injury. Fourth, the lack of clinical data, such as disease severity and duration, might miss some factors affecting myocardial injury. Therefore, in the future, a well-designed prospective study is necessary to verify the conclusions of the present study.

## 5. Conclusions

In our study, we provided evidence that myocardial injury is not unusual in patients with MG. Additionally, older age, infection, myasthenic crisis, thymoma, and ICI therapy were independently associated with myocardial injury in MG patients. Additionally, we found a close association between myocardial injury and death. Therefore, the timely measurement of TnT levels may be beneficial. Careful attention should be paid to myocardial injury in MG patients.

## Figures and Tables

**Table 1:** Sex-specific baseline characteristics of MG patients with normal and elevated TnT levels (n = 336).

	Male		*p* Value	Female		*p* Value
	Normal TnT	Elevated TnT		Normal TnT	Elevated TnT	
Number	60	88		84	104	
Age, mean (SD), years	51.9 (16.6)	54.7 (17.1)	0.324	49.2 (17.2)	56.0 (16.6)	0.007
Infection, n (%)	18 (30)	50 (56.8)	0.001	27 (32.1)	68 (65.4)	<0.001
Myasthenic crisis, n (%)	8 (13.3)	33 (37.5)	0.001	15 (17.9)	35 (33.6)	0.015
Classification						
Available, n	59	86		83	100	
Generalized MG, n (%)	42 (71.2)	71 (82.6)	0.105	68 (81.9)	91 (91.0)	0.070
Ocular MG, n (%)	17 (28.8)	15 (17.4)		15 (18.1)	9 (9.0)	
Thymoma, n (%)	17 (28.3)	30 (34.1)	0.460	14 (16.7)	40 (38.5)	0.001
Atrial fibrillation	0	1 (1.1)	0.407	0	4 (3.9)	0.069
ICI therapy, n (%)	1 (1.7)	3 (3.4)	0.521	0	3 (2.9)	0.117
Outcome						
Death, n (%)	0	21 (23.9)	<0.001	0	16 (15.4)	<0.001

Abbreviations: MG, myasthenia gravis; TnT, troponin T; SD, standard deviation; ICI, immune checkpoint inhibitor; and p, *p* values of comparisons between groups according to *t* test and Pearson’s chi-squared test.

**Table 2:** Comparisons of echocardiographic measurements between patients with normal and elevated TnT levels.

	Male		*p* Value	Female		*p* Value
	Normal TnT	Elevated TnT		Normal TnT	Elevated TnT	
LAD	31.8 (4.8)	31.1 (6.9)	0.618	29.4 (7.4)	28.4 (9.3)	0.579
LVEDD	46.1 (3.6)	46.6 (3.7)	0.591	44.6 (3.2)	42.9 (3.4)	0.017
RVEDD	20.7 (4.3)	20.6 (4.3)	0.916	19.3 (4.7)	18.9 (3.6)	0.678
RAD	32.5 (6.7)	30.9 (8.6)	0.394	28.4 (10.6)	28.7 (10.3)	0.902
IVS	10.2 (2.7)	10.5 (2.4)	0.664	8.9 (2.1)	9.7 (3.4)	0.219
LVPW	8.8 (1.9)	8.8 (2.3)	0.999	7.8 (2.2)	8.4 (2.7)	0.269
LVEF	67.0 (5.5)	65.7 (8.6)	0.462	64.6 (14.6)	68.2 (4.2)	0.127

Note: the data were displayed as mean (SD) millimeters. Abbreviations: LAD, left atrial diameter; LVEDD, left ventricular end-diastolic diameter, RVEDD, right ventricular end-diastolic diameter; RAD, right atrial diameter; IVS, interventricular septum thickness; LVPW, left ventricular posterior wall thickness; and LVEF, left ventricular ejection fraction.

**Table 3:** Univariate and adjusted multivariate linear regression models with log TnT levels as the dependent variable were performed for MG patients with available TnT levels.

	Unadjusted		Adjusted	
	Coefficient (95% CI)	*p*	Coefficient (95% CI)	*p*
Age	0.004 (0.000, 0.008)	0.029	0.004 (0.000, 0.007)	0.034
Female	−0.073 (−0.206, 0.059)	0.278	−0.063 (−0.182, 0.056)	0.301
Infection	0.371 (0.245, 0.497)	<0.001	0.240 (0.097, 0.382)	0.001
Myasthenic crisis	0.453 (0.312, 0.593)	<0.001	0.312 (0.158, 0.466)	<0.001
Generalized MG	0.249 (0.072, 0.425)	0.006	−0.012 (−0.181, 0.158)	0.888
Thymoma	0.206 (0.064, 0.348)	0.005	0.228 (0.097, 0.360)	0.001
ICI therapy	1.218 (0.775, 1.662)	<0.001	1.220 (0.812, 1.628)	<0.001

Abbreviations: MG, myasthenia gravis; TnT, troponin T; CI, confidence interval; and ICI, immune checkpoint inhibitor.

**Table 4:** Comparisons of the clinical characteristics of the dead and surviving MG patients with available troponin T levels.

	Dead Patients	Surviving Patients	*p*
Number	37	299	--
Female, n (%)	16 (43.2)	172 (57.5)	0.099
Age, mean(SD), years	58.2 (16.0)	52.6 (17.1)	0.060
Infection, n (%)	34 (91.9)	129 (43.1)	<0.001
Thymoma, n (%)	19 (51.4)	82 (27.4)	0.003
ICI therapy, n (%)	2 (5.4)	5 (1.7)	0.134
Myasthenic crisis, n (%)	37 (100.0)	54 (18.1)	<0.001
Classification			
Available, n	37	291	--
Generalized MG, n (%)	37 (100.0)	235 (80.8)	0.003
Ocular MG, n (%)	0	56 (19.2)	--

Abbreviations: MG, myasthenia gravis; SD, standard deviation; and ICI, immune checkpoint inhibitor.

**Table 5:** Univariate and adjusted multivariate logistic regression models with death as the dependent variable were performed for MG patients with available TnT levels.

	Unadjusted		Adjusted	
	Odds Ratio (95% CI)	*p*	Odds Ratio (95% CI)	*p*
Log TnT levels	7.928 (4.388, 14.325)	<0.001	8.818 (4.107, 18.934)	<0.001
Age	1.020 (0.999, 1.042)	0.062	1.028 (0.994, 1.063)	0.112
Female	0.562 (0.282, 1.121)	0.102	0.346 (0.139, 0.862)	0.023
ICI therapy	3.360 (0.628, 17.972)	0.157	0.514 (0.042, 6.308)	0.603
Thymoma	2.793 (1.397, 5.585)	0.004	5.092 (1.861, 13.938)	0.002
Infection	14.935 (4.488, 49.708)	<0.001	14.597 (3.345, 63.700)	<0.001

Abbreviations: MG, myasthenia gravis; TnT, troponin T; ICI, immune checkpoint inhibitor; and CI, confidence interval.

## Data Availability

Anonymized data not published within this article will be made available to any qualified investigator, by directly requesting from corresponding author.
